# Development of a Vascularized Human Skin Equivalent with Hypodermis for Photoaging Studies

**DOI:** 10.3390/biom12121828

**Published:** 2022-12-07

**Authors:** Martina M. Sanchez, Thamidul Islam Tonmoy, B. Hyle Park, Joshua T. Morgan

**Affiliations:** Department of Bioengineering, University of California, Riverside, CA 92521, USA

**Keywords:** HSE, human skin equivalent, tissue engineering, self-assembly, scaffold, aging, photoaging

## Abstract

Photoaging is an important extrinsic aging factor leading to altered skin morphology and reduced function. Prior work has revealed a connection between photoaging and loss of subcutaneous fat. Currently, primary models for studying this are in vivo (human samples or animal models) or in vitro models, including human skin equivalents (HSEs). In vivo models are limited by accessibility and cost, while HSEs typically do not include a subcutaneous adipose component. To address this, we developed an “adipose-vascular” HSE (AVHSE) culture method, which includes both hypodermal adipose and vascular cells. Furthermore, we tested AVHSE as a potential model for hypodermal adipose aging via exposure to 0.45 ± 0.15 mW/cm^2^ 385 nm light (UVA). One week of 2 h daily UVA exposure had limited impact on epidermal and vascular components of the AVHSE, but significantly reduced adiposity by approximately 50%. Overall, we have developed a novel method for generating HSE that include vascular and adipose components and demonstrated potential as an aging model using photoaging as an example.

## 1. Introduction

Human skin provides essential physical protection, immune barrier function, and thermal regulation [1]. As humans age, there is a decline of skin function, including loss of barrier function and healing capacity [2]. This correlates with structural changes including decreased vasculature, decreased dermal elasticity and collagen organization, stiffening, lower hydration, and reduced dermal and hypodermal (or subcutaneous fat) volumes [3,4,5,6,7,8,9,10,11,12,13,14,15,16,17]. These detrimental effects of natural aging are compounded by extrinsic aging factors including ultraviolet A (UVA; 320–400 nm) [18] photoaging that occurs with sun exposure [2,19,20,21]. With normal aging, the skin is smooth with fine wrinkles and has decreasing elasticity. With photoaging skin is coarse, rough, even lower elasticity, and has changes in pigmentation [9,17]. In particular, UVA sun exposure mainly damages the generation of reactive oxygen species [18,22], The primary effects are in the dermis and hypodermis, with the epidermis being primarily damaged by UVB. Furthermore, UVA exposure to human skin has demonstrated decreased expression of subcutaneous adipokines, such as adiponectin [23,24,25]. These effects are harmful since adipokines have been found to benefit wound healing and anti-inflammatory skin properties. The hypodermis as a whole contributes to thermal regulation, skin elasticity, and regeneration [26,27]. UVA exposure additionally degrades the dermal matrix through decreases in procollagen synthesis and increases in MMP-1, -3, and -9 expression [23,24,25]. Photoaged skin has also exhibited reduced dermal vasculature and dermal connective tissue breakdown and disorganization in human explant cultures [3,18,25,28,29,30].

Human Skin Equivalents (HSEs) are in vitro tissue models that have been previously used for studies on photoaging, wound healing, skin development, alopecia, disease, stem cell renewal, and toxicology screening research [31,32,33,34,35,36,37,38,39,40,41,42,43,44,45,46,47,48,49,50]. The models rely on self-assembly of skin components within an appropriate matrix. Traditionally-used animal models, such as rabbits, pigs, mice, and rats, have different physiology than humans, for example in wound healing [31,51]. These examples add to longstanding recognition of the limitations inherent to animal models [52], and bolster recent consideration of reduction strategies [53,54]. While HSEs present with limitations of their own, they are increasingly demonstrated as useful models for human skin [34,35,36,37,38].

Although HSE research has been well developed to recreate the dermal and epidermal layers using fibroblasts and keratinocytes, novel co-culture systems are needed to recapitulate human anatomy more closely [55] and mimic trophic factor exchange of different cell populations in vivo [40,55,56,57,58]. Building on our previously published protocol generating vascularized HSE (VHSE) [59,60], here we demonstrate inclusion of a hypodermis, which we term adipose and vascular human skin equivalent (AVHSE), and demonstrate suitability for UVA photoaging studies. Multi-cellular skin models similar to this AVHSE have been previously explored but with fewer cell types, much shorter culture lengths, and little to no volumetric characterization [61,62,63,64]. UV photoaging has been previously investigated with in vitro skin models of the epidermis [50], keratinocytes in 2D [65], dermal fibroblasts in 2D [66,67], and adipose components in 2D [24,26]. This work combines photoaging studies with comprehensive in vitro skin models and allows for volumetric quantification of epidermal, dermal, and hypodermal components through volumetric imaging (confocal and optical coherence tomography). Furthermore, the effects of photoaging on adipokine and inflammatory cytokines have been quantified using ELISA.

## 2. Materials and Methods

### 2.1. Cell Culture

AVHSE cultures were created using a modified form of our prior VHSE protocol [59,60]. Briefly, N/TERT1 human keratinocytes (hTERT immortalized; gift of Dr. Jim Rheinwald and Dr. Ellen H. van den Bogaard [31,68]), HMEC1 human microvascular endothelial cells (SV40 immortalized; ATCC, Manassas, VA; #CRL-3243) [69], and primary adult human dermal fibroblasts (HDFa; ATCC #PCS-201-012) were used as previously described, and ASC52telo adipose derived mesenchymal stem cells (hTERT immortalized; ATCC #SCRC-4000) [70] were included for the hypodermis. All cell lines were routinely cultured at 37 °C and 5% CO_2_; all media blends given in Table 1**.** N/TERT1 cells have been shown to maintain normal epidermal behavior in previous organotypic skin cultures [31,35,68]. N/TERT1 cells (passages: 8,10,16,19) were grown up in a modified K-SFM media blend including K-SFM base, 0.2 ng/mL endocrine growth factor (EGF), 25 µg/mL bovine pituitary extract, 0.3 mM CaCl_2_, and 1% penicillin/streptomycin (PCN/STREP). N/TERT1 were routinely passaged once 30% confluence was met to prevent undesired differentiation in 2D cultures [68]. HMEC1 cells were grown up in MCDB1 base media with 10 mM L-glutamine, 1 µg/mL hydrocortisone, 10 ng/mL EGF, 10% FBS, and 1% PCN/STREP. HMEC1 cells at passages 9 and 11 were used. HDFa were originally expanded in fibroblast basal media supplemented with fibroblast growth kit per manufacturer instructions. For short term expansion immediately prior to AVHSE cultures, HDFa cells (all passage 4) were grown up in DMEM (4.5 g/L glucose) supplemented with 5% FBS and 1% FBS. ASC52telo were used to generate the adipose component of the skin construct. Cells were originally expanded in mesenchymal stem cell basal media (ATCC #PCS-500-030) with added supplements from a mesenchymal stem cell growth kit (ATCC #PCS-500-040) and G418 at 0.2 mg/mL; this was used as the 2D culture media until adipogenesis induction. Adipogenesis media [71,72] (recipe given in Table 1) was administered once ASC52telo plates were ~90% confluent (ASC52telo passages: 6, 8, and 10 were used for AVHSEs).

### 2.2. Collagen Isolation: Rat-Tail Collagen

Collagen Type I was isolated from rat tail tendons as described previously [79,80,81,82]. Briefly, tendons were extracted from rat tails (Pel-Freez Biologics, Rogers, AR, USA), washed in Dulbecco’s phosphate buffered saline (PBS) and soaked in acetone for 5 min, 70% isopropanol for 5 min, then dissolved in 0.1% glacial acetic acid for at least 72 h rocking at 4 °C. After dissolving, collagen was centrifuged at ~20,000× *g* for 1 h and the supernatant was frozen at −80 °C and lyophilized for long term storage at −80 °C. When ready to use, collagen was dissolved in 0.1% glacial acetic acid to 8 mg/mL and stored at 4 °C.

### 2.3. Construct Fabrication Overview

**Figure 1:**
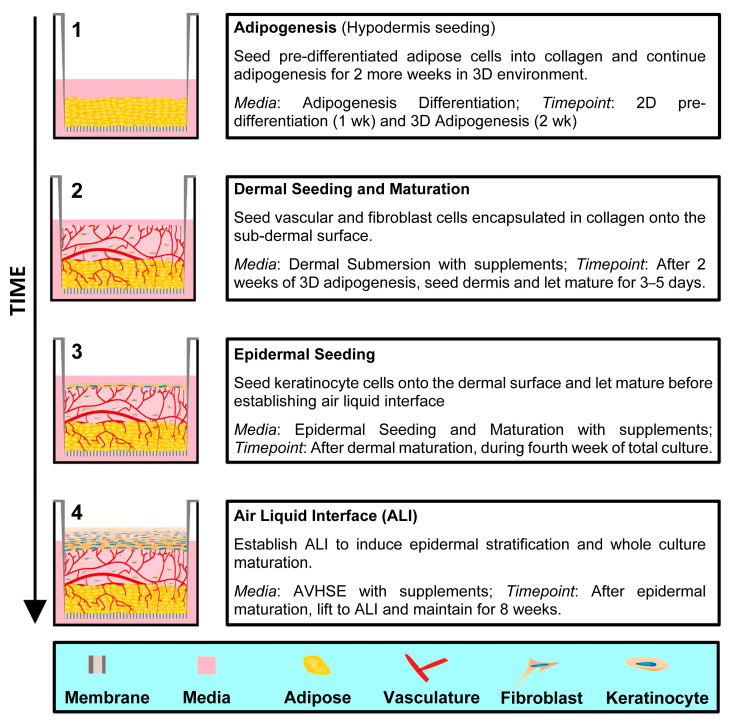
AVHSE generation. There are four main steps in creating an AVHSE: (1) Adipogenesis, (2) Dermal seeding and maturation, (3) Epidermal seeding, and (4) Air liquid interface. Cartoons on the left show cross-sectional representations of AVHSE during each step.

Generation of AVHSE cultures includes four main steps, shown graphically in Figure 1: (1) adipogenesis, (2) dermal seeding and maturation, (3) epidermal seeding, and (4) air liquid interface (ALI); total duration is approximately twelve weeks: adipose differentiation (3 weeks), dermal maturation (<1 week), epidermal seeding (2–3 days), air liquid interface exposure (8–9 weeks). Collagen gel was used to create the hypodermis and dermal layer of the AVHSE constructs in 12-well culture inserts (translucent PET, 3 µm pore; Greiner Bio-One, Monroe, NC; ThinCerts #665631), similar to previously used [31,35,36,37,38,42,43]. In all cases, final collagen concentration was 3 mg/mL [59].

### 2.4. Adipogenesis and Hypodermal Seeding

ASC52telo cells were grown to >90% confluent and adipogenesis was induced for 3 weeks (media blend given in Table 1), split between 1 week in 2D culture and 2 weeks in 3D culture. For 3D culture, ASC52telo cells (750,000 cells/mL of collagen) were encapsulated in 125 µL of 3 mg/mL collagen and seeded into the culture insert. After gelation, constructs were submerged with adipogenesis media (~0.5 mL and ~1 mL of media in the culture insert and well, respectively). Media was added to the insert chamber first to prevent detachment of the collagen from the membrane). Media was changed every 2–3 days until dermal seeding.

### 2.5. Dermal/Epidermal Seeding and Air Liquid Interface

Media was aspirated from each well and 250 µL of 3 mg/mL collagen with HMEC1 and HDFa cells (750,000 and 75,000 cells/mL of collagen, respectively) was seeded onto the hypodermis, then quickly transferred to 37 °C for gelation. After gelation, constructs were submerged with dermal submersion (DS) media (Table 1) supplemented with 3% FBS, 2 ng/mL vascular endothelial growth factor (VEGF-A; Peprotech, Cranbury, NJ, USA; #100-20) and 100 µg/mL L-ascorbic acid (L-AA; Thermo Fisher Scientific, Waltham, MA, USA). Media was changed every 2–3 days with fresh L-AA [59,79,83]. After 3–5 days of growth in submersion, DS media was aspirated and epidermal seeding and maturation media (ESM) supplemented with 1% FBS and 100 µg/mL L-AA (1.5 mL added to each well). N/TERT1 keratinocyte cells were immediately seeded dropwise at 170,000 cells per insert (~1.13 cm^2^ growth area) using 200 µL of their maintenance media, K-SFM. One/two days after epidermal seeding, media was changed to AVHSE media and the cultures were lifted to ALI within 8–24 h, with longer times leading to increased contraction [59]. The process to establish ALI was outlined previously [59]; typical ALI was established with ~1 mL of media. Following ALI establishment, media was changed every 2–3 days with AVHSE media and supplemented with 100 µg/mL L-AA and 30 nM selenium (sodium selenite; ThermoFisher Scientific, Waltham, MA, USA).

### 2.6. Photoaging of AVHSEs

After completing 7 weeks at ALI, AVHSEs were exposed to UVA to model photoaging (PA). A UVA LED array was established by drilling a 5 mm through-hole at center of each well in the plate lid and inserting a 385 nm/80 mcd LED (VAOL-5GUV8T4; VCC, Carlsbad, CA, USA); 1 LED directly illuminated each insert (Figure S1). Four LEDs were powered in series with ~10 mA, providing a 0.45 ± 0.15 mW/cm^2^ dose as measured by a UV sensor (UVAB Digital Light Meter, #UV513AB, General Tools & Instruments, Secaucus, NJ, USA). LEDs were measured every 3–4 days and replaced as needed. AVHSEs were exposed to UVA for 2 h daily for one week using an automated timer. UV dose and exposure was determined within values of prior work on human skin equivalents, cell monolayers, and mouse models [18,23,24,26,50,84,85].

### 2.7. ELISA (Adiponectin, IL-6, and MMP-1)

AVHSE culture supernatant was collected at the end of ALI week 8 from controls and photoaged samples. Samples were centrifuged and frozen at −80 °C until use. ELISAs were performed for human Adiponectin, Interleukin-6 (IL-6), and total matrix metalloproteinase (MMP-1) according to the manufacturer’s protocol (Proteintech Group, Rosemont, IL, USA). Each sample was assayed in duplicate with standards completed for each run. For color development, tetramethylbenzidine (TMB)-substrate exposure was 20 min for Adiponectin and IL-6, and 15 min for MMP-1 at 37 °C in the dark. After stop solution was administered, color development was immediately measured at 450 nm with a correction wavelength of 630 nm using a SpectraMax M2 Multi-mode microplate reader (Molecular Devices, San Jose, CA, USA) and corrected against a run zero standard. Four parametric logistic curves (4PLC) fits were used and values below detection limit were set to zero. Sample sizes varied for each assay due to sample availability and are as follows: Adiponectin: for each condition *n* = 10, r^2^ = 0.9975; IL-6: control *n* = 12 and photoaged *n* = 8, r^2^ = 0.9993; MMP1: control *n* = 7 and photoaged *n* = 10, r^2^ = 0.9954 (9 values) and 0.91 (7 values).

### 2.8. Post-Culture Immunostaining and Confocal Microscopy

After culture, samples were pre-fixed in 4% paraformaldehyde for 5 min then fixed for 1 h in 4% paraformaldehyde and 0.5% Triton ×100 at room temperature. Samples were washed three times in PBS then stored at 4 °C until staining. For staining, culture insert membranes were removed using forceps, as described previously [59]. The staining and imaging processes were completed in four phases: epidermal, dermal vasculature, adipose, and post-clearing (Table 2). The nuclear marker DRAQ7 was administered during the epidermal staining phase and was used until imaging was completed. Imaging orientation of the AVHSEs were dependent on stain phase (Table 2). For staining, primary and secondary antibody stain solutions were made up in blocking buffer (Table 2). All samples were stored at 4 °C in PBS until imaging.

To image each fixed sample, custom polydimethylsiloxane (PDMS; Dow Corning, Midland, MI, USA) molds were punched specific to each sample size and adhered to glass slides [59]. Samples were placed in the well with PBS and covered with another glass slide to preserve humidity while imaging. As AVHSE are too thick for direct confocal imaging throughout the structure without tissue clearing, each sample was imaged in both apical and basal orientations. Stains were multiplexed to laser excitations in cases of minimal overlap (e.g., epidermal and subdermal stains), and this was confirmed through the sequential staining process.

### 2.9. Tissue Clearing

After completing staining and imaging phases 1–3, constructs were cleared via methyl salicylate with methanol dehydration. Constructs were dehydrated in methanol with 4 × 10 min baths then cleared in methyl salicylate with 4 × 5 min baths. Constructs were stored in methyl salicylate and imaged via confocal microscopy on the same day, as detailed previously [59,60].

### 2.10. Quantitative Epidermal Analysis

Thickness of epidermal layers were automatically detected from confocal images via thresholding differences using a custom analysis algorithm designed in MATLAB (MATLAB 2018b; Mathworks, Natick, MA, USA), similar to prior descriptions [60]. For each sample, five confocal sub-volumes in the center of the AVHSE were used to detect thickness (total volume of 1.85 × 0.37 × 0.25–0.4 mm; imaging depths were adjusted per sample but a consistent voxel size of 0.7 × 0.7 × 3 µm was used). An average thickness was found for each XY position to obtain a volumetric thickness indication rather than from a single cross-sectional position or from max projection. Briefly, epidermis was localized using DRAQ7, cytokeratin 10, and involucrin stains. Noise was removed using median filters applied to each XY-plate and intensities were scaled by linear image adjustment. Background auto-fluorescence was removed using rolling ball filters on each XY plane and the epidermis was segmented using hysteresis thresholding. Gaps in the epidermal binary volume were removed via morphological closing and opening with a disk structuring element. The resulting binary volume created a computational plane from which the top and bottom difference could be calculated and metrically scaled by appropriate voxel size. Intensity comparison of the suprabasal markers, Cytokeratin 10 and Involucrin, was completed across all samples using confocal images. A maximum projection image of ten positions per sample was generated and average intensity values were calculated. For all epidermal quantification, five AVHSE replicates were used for analysis.

### 2.11. Quantitative Dermal/Hypodermal Analysis

Adipose thickness, volume fraction (VF), and integrated intensity quantification were completed from 10 confocal sub-volumes per each sample (a total volume of 3.7 × 0.37 × 0.35 mm). VF is an estimate of the adipose within the hypodermis and dermal space. Volumetric thickness was calculated using localization of the BODIPY mature adipose marker, as described for epidermal thickness quantification. Integrated intensity of BODIPY was quantified via custom algorithms. Briefly, image sub-volumes were segmented and the resulting binary masks were used to isolate BODIPY stain from background noise and autofluorescence. The sum of raw intensity along the z-axis was calculated for each sub-volume within its binary map, then all sub-volume values were averaged as a metric of the whole sample volume. These data were gathered from images taken in the 3rd imaging phase (Table 2). Six AVHSE replicates were used for analysis.

Vascular quantification parameters of diameter, VF of the vasculature, and diffusion length (R_k_) were determined from the average of 6 confocal sub-volumes per each sample (total volume of 2.22 × 0.37 × 0.35 mm), similar to published methods [59,79,86]. Using the Collagen IV marker from cleared AVHSE structures (4th imaging phase, Table 2), vessels were located through segmentation (using built-in MATLAB functions, custom functions, and previously published functions [87,88]), and, ultimately, vessel detection via an enhanced Hessian based Frangi filter [89,90,91]. VF was determined using the resulting volume segmentation. After locating vessels, the segmented volume was skeletonized through a fast marching algorithm [59,79,92,93,94]. Diameter was quantified by performing Euclidean distance transform on the vascular segmentation and collecting values along the skeleton. Additionally, R_k_ was defined as a “diffusion length” from the vascular fraction that encompasses 90% of the volume [79]. R_k_ was obtained by determining the Euclidean distance between all points in the collagen volume and the nearest point on the network. Four AVHSE replicates each were used for analysis.

### 2.12. Live Culture Imaging

On a limited number of cultures, optical coherence tomography (OCT) was used as a non-invasive technique to measure epidermal thickness in live samples as previously described [60]. OCT imaging was conducted with a custom built fiber-based spectral domain optical coherence tomography (SD-OCT) system centered at 1310 nm, as described previously [95]. Each sample was imaged then immediately returned to culture while maintaining sterility, requiring imaging through the well plate lid. To minimize the reflective effect of the lid, the sample arm of the OCT system was tilted at 15°, reducing reflection while maintaining adequate illumination. Imaging took ~1 h for each sample; no loss of sample viability was observed. Settings for imaging remained consistent through culture: 1 volume, 400 frames, and 4096 A-lines were taken per sample; resulting image size was 4096 × 512 × 400 voxel (4 × 2 × 4 mm). Epidermal thickness was assessed via post-processing of the data using custom-written scripts in MATLAB (MATLAB 2018b; Mathworks, Natick, MA, USA), which detected the top and bottom surfaces of the hyper-reflective epidermis and calculated thickness across the volume, as previously described [60].

### 2.13. Statistics

Pairwise comparisons of control v. photoaged samples were completed through a two-tailed *t*-test. ANOVA followed by Tukey’s HSD post hoc test was used to test for statistically significant differences when applicable. Un-normalized data points are shown for comparison to tissue scale morphology. For statistical comparison, data were normalized to control for epidermal, vascular, and adipose quantification. Significant differences of normalized data are plotted with *p* < 0.05 represented by a single asterisk; *p* < 0.01 represented by a double asterisk.

## 3. Results

### 3.1. AVHSE Enables Tissue-Scale Studies of Skin Biology

**Figure 2:**
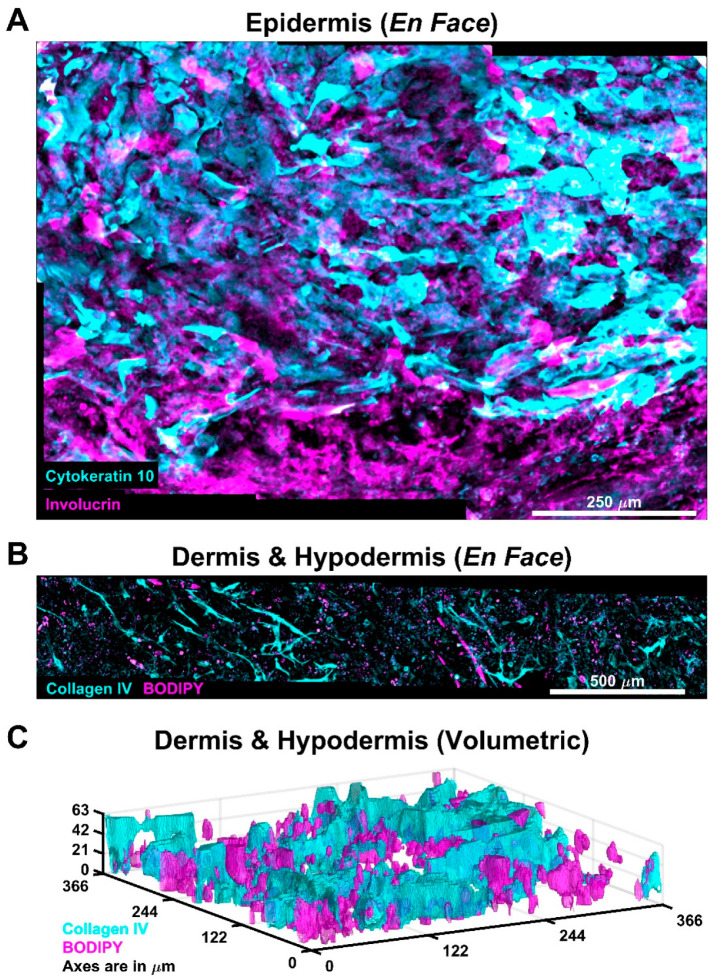
AVHSE allow for large-scale assessment of cultures. (**A**) Demonstration of scale of epidermal analysis. Cytokeratin 10 (cyan) is a suprabasal epidermal marker and Involucrin (magenta) is a stratum corneal marker. Image is an en face max projection of ~0.7 × 1 × 0.2 mm volume. (**B**) Adipose and vasculature morphology can be assessed at scales that span 3.6 mm (approximately half of this representative AVHSE), presented as an en face max projection. Collagen IV (cyan) marks the vasculature and BODIPY (magenta) marks lipid droplets secreted from mature fat cells (magenta). (**C**) Volumetric rendering of segmented Collagen IV (cyan) and BODIPY (magenta), demonstrating vascular infiltration into the hypodermis. Images were acquired pre-clearing and are median filtered for clarity.

AVHSEs and the analysis techniques presented here enable study of skin volumetrically and at the tissue scale (Figure 2). Through automated imaging and stitching, epidermal, dermal, and hypodermal markers can be assessed volumetrically. The automated image analysis of the three skin compartments described in the following sections was completed on biologically large volumetric areas with minimum volumes of ~1 × 0.7 × 0.25 mm to analyze the epidermis and up to 3.6 × 0.37 × 0.35 mm to analyze the hypodermis. Importantly, the volumetric approach allows assessment of variation across the culture that is difficult with standard histological approaches that involve sectioning [60].

### 3.2. UVA Photoaging Alters Adiponectin Expression

**Figure 3:**
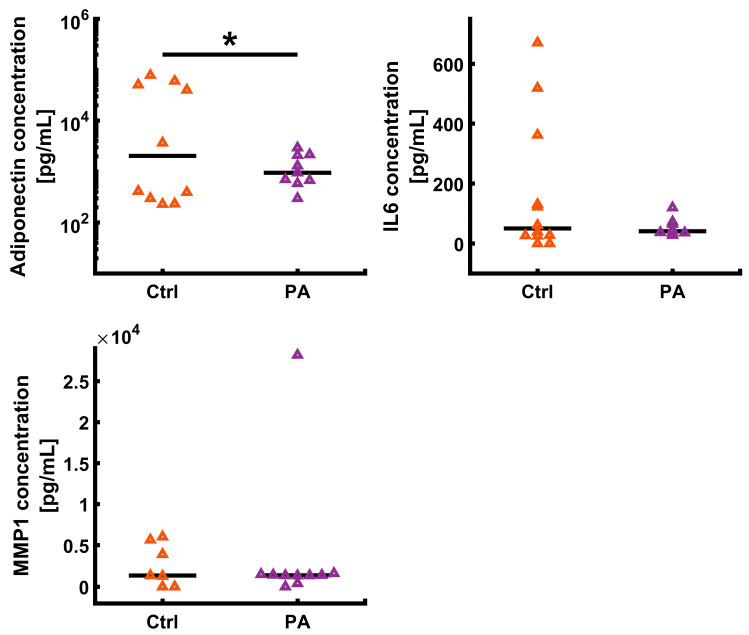
Cytokine evaluation from cell media was completed via ELISA. Cell media was collected after week 8 of culture. All values were corrected by a zero standard and values below detection limit were set to zero. All values were determined from four-parametric logistic curve fits. Data is shown as medians (black bars) and individual data points (triangles). Sample numbers varied for each assay due to limited culture volumes. Adiponectin: for each condition *n* = 10. IL-6: control *n* = 12 and photoaged *n* = 8; MMP1: control *n* = 7; and photoaged *n* = 10. A two-tailed *t*-test showed a significant decline in the adiponectin secreted into media after photoaging AVHSEs (*p* < 0.05; indicated with *).

Prior studies have demonstrated decreased adipokine production during photoaging, and adipokines are mediators of the dermal photoaging mechanism [24,96]. To test if the AVHSE cultures were similarly responsive to UVA, we measured production of adiponectin using ELISA. AVHSE cultures were prepared and maintained through ALI as described in the methods. After 7 weeks of ALI, AVHSE were exposed to 7 days of UVA (2 h/day, 385 nm, 0.45 ± 0.15 mW/cm^2^), or left as controls. Media supernatant was collected from both photoaged and control samples after UVA exposure. Adiponectin expression was significantly reduced, in agreement with prior in vivo studies [24] (Figure 3). This was not accompanied by a general inflammatory response or increased matrix metalloproteinase-1 (MMP-1) presence, as indicated by stable IL-6 and MMP-1 expression (Figure 3).

### 3.3. Epidermis Is Stable during UVA Photoaging

**Figure 4:**
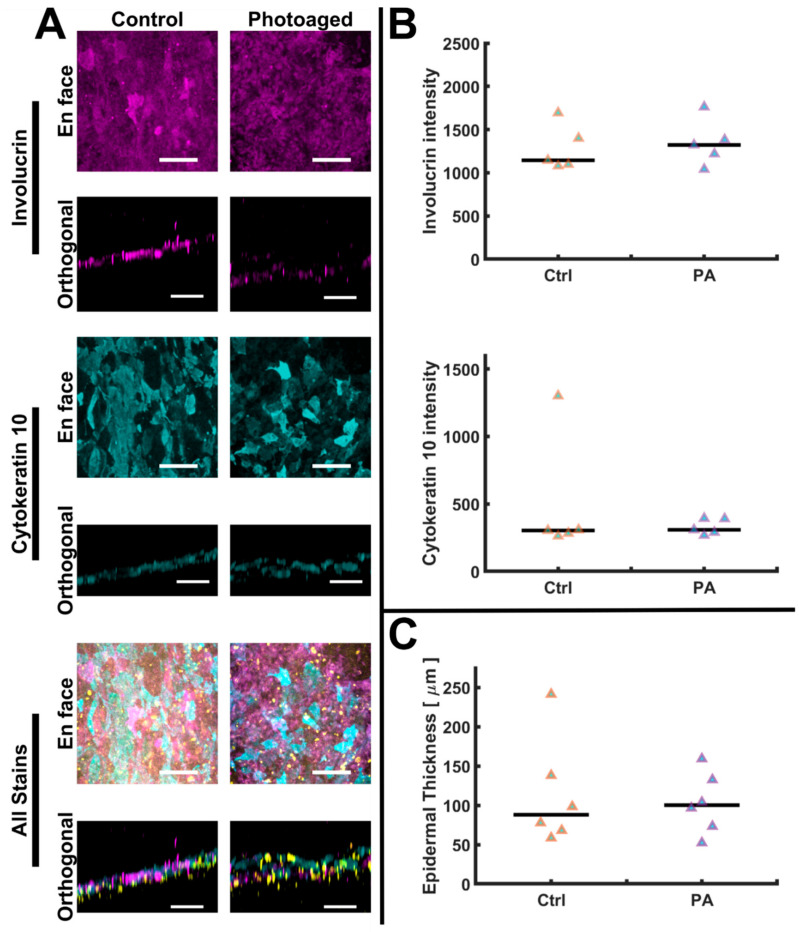
Epidermal characterization and quantification. (**A**) The epidermal differentiation markers, Involucrin (magenta) and Cytokeratin 10 (cyan), localize to epidermis. Nuclei are marked with a DRAQ7 counterstain and shown in yellow. No apparent qualitative changes in the experimental groups were observed, as shown in these representative images. Scalebars are 100 µm. (**B**) Quantification of epidermal intensities was completed from z-axis maximum projections; no indication of intensity changes was found in either epidermal stain when comparing control (Ctrl) to photoaged (PA) samples. For both control and photoaged groups *n* = 5. (**C**) Epidermal thickness was volumetrically quantified and no differences were indicated (*n* = 6 for both control and photoaged groups). Data is shown as medians (black bars) and individual data points (triangles). Images were acquired pre-clearing and are median filtered for clarity.

Photoaging by the UVA largely acts on the dermal and hypodermal portions of the skin rather than the epidermis, in contrast to UVB, which shows epidermal toxicity [18,22]. To assess any changes in epidermal morphology, we stained suprabasal markers (involucrin and cytokeratin 10) along with the nuclear stain DRAQ7 to assess epidermal thickness. No statistically significant differences were observed in the staining intensity of involucrin and cytokeratin 10 (Figure 4A,B), or in the overall thickness of the epidermis (Figure 4C), when comparing the control and the photoaged AVHSEs. For a limited number of samples, we further assessed the epidermis through OCT imaging as previously described [60]. Consistent with the confocal data we observed no gross change in epidermal morphology with photoaging. These data are consistent with the minimal in vivo effects of UVA on the epidermis [22].

### 3.4. Dermal Vasculature Is Stable during UVA Photoaging

**Figure 5:**
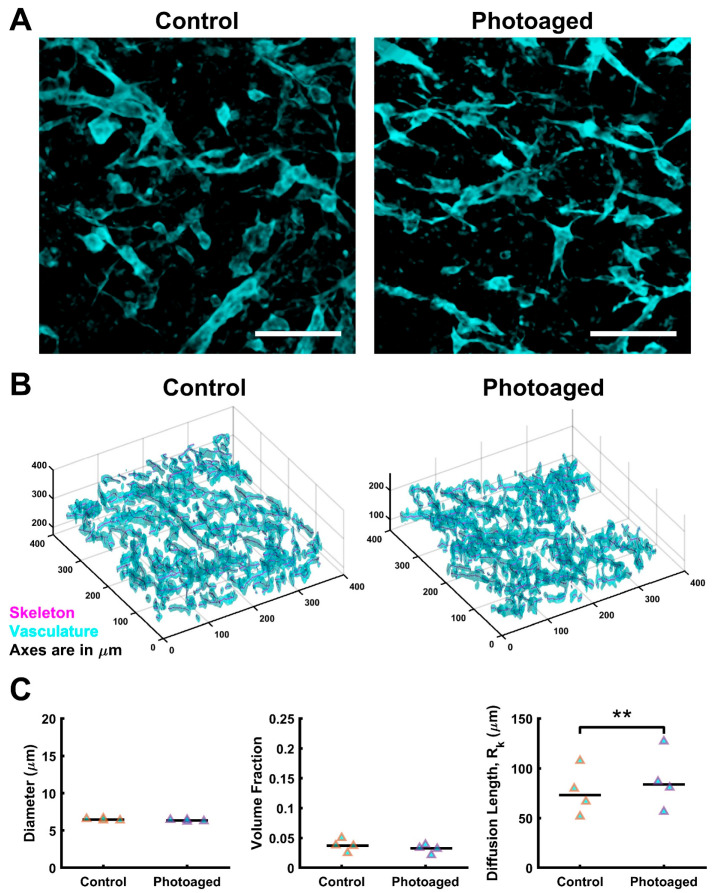
Vascular staining and quantification. (**A**) A comparison of maximum projections of confocal images of control v. photoaged AVHSE sub-dermis and dermis; Collagen IV marks vasculature in cyan. Scalebars are 100 µm. (**B**) Segmentation of the vascular fraction (cyan) was completed on 6 cleared sub-volumes per sample. Skeletonization was completed using segmentation data (magenta line). Shown is a representative 3D rendering of one confocal sub-volume. (**C**) Segmentation and skeletonization of vascular networks enable quantitative assessment of the morphology (*n* = 4 for each condition). Vessel diameter and volume fractions remain stable when AVHSEs are photoaged and there is an increase in diffusion length for photoaged (*p* < 0.01; indicated with **). Data is shown as medians (black bars) and individual data points (triangles). Images are median filtered for clarity.

Prior studies have shown dermal vascular damage is associated with chronic UVA exposure, as determined from sun-exposed skin biopsies from young v. aged individuals (20–80 years) [97]. As a proxy for vascular damage, we quantified overall morphology in the AVHSE. Vascular structures were identified through localization of collagen IV (Figure 5A). Formation of well-developed vascular networks was observed in both control and photoaged samples, as shown in maximum projections. Imaging for vascular quantification was performed after tissue clearing, to minimize the loss of signal deeper in the confocal volume. The 3D rendering shown is representative of the vascular network segmentation and skeletonization that was made possible with cleared tissues (Figure 5B). Vascular network diameters were quantified as 6.45 ± 0.14 μm for control and 6.34 ± 0.12 μm for photoaged (median ± S.E.M.). Volume fraction (VF) of vasculature had median values of 0.037 ± 0.01 and 0.032 ± 0.007 (control and photoaged, respectively; median ± S.E.M.). No statistical difference was determined in comparison of diameter or vascular VF. Diffusion length (R_k_) [79] was calculated with median values of 73.16 ± 23.75 and 83 ± 29.36 microns (control and photoaged, respectively; median ± S.E.M.). A significant increase in diffusion length of photoaged AVHSEs was detected (*p* < 0.01; normalized to biological replicate controls) which corresponds to a slight non-significant decrease in VF of photoaged samples, consistent with slight loss of vascular density.

### 3.5. Hypodermal Adiposity Is Reduced with Photoaging

**Figure 6:**
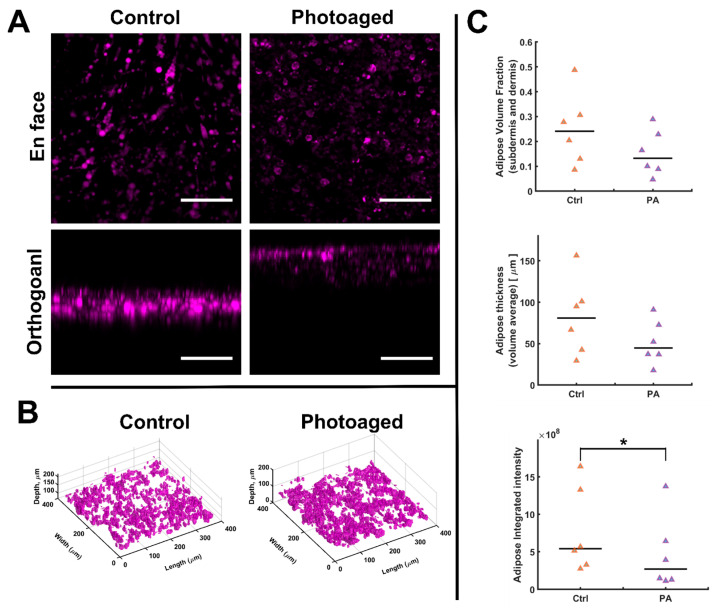
Adipose staining and quantification. (**A**) A comparison of confocal images of control vs. photoaged AVHSE sub-dermis; BODIPY marks lipid accumulation at mature adipocytes in magenta. Scalebars are 100 µm. (**B**) Representative 3D rendering of adipose volume fraction for control and photoaged samples. (**C**) Top-Volume Fraction was calculated based on segmentation of the BODIPY stain as seen in B. Middle-Thickness quantification based on morphological closing of BODIPY segmentation. Bottom-Integrated Intensity of BODIPY across the volume shows a significant (*p* < 0.05; indicated with *) drop in photoaged samples (*n* = 6 for each condition). Data is shown as medians (black bars) and individual data points (triangles). Images were acquired pre-clearing and are median filtered for clarity.

Prior in vivo studies have shown decreases in hypodermal adipose associated with UVA photoaging. To test if this was mimicked in the AVHSE model, we used confocal imaging of the lipid stain BODIPY in both controls and photoaged AVHSE. Representative images shown in Figure 6A show decreased staining intensity and representative volume renderings are shown in Figure 6B. To quantify adiposity, we utilized two morphological measures (lipid volume fraction and adipose thickness), and the integrated intensity of the BODIPY. Both morphological measures exhibit subtle declines, but the results are non-significant (Figure 6C). However, the overall stain intensity was significantly decreased (Figure 6C), indicating an overall loss of lipid content in the photoaged AVHSE.

## 4. Discussion

Skin provides a critical barrier, insulation, and homeostatic functions in human physiology; these are known to be disrupted in aging [2,17,98]. Despite the importance, research is limited by the accessibility of physiologically relevant models, with conventional culture methods lacking the structure and organization of the overall tissue [4] and conventional animal models presenting key differences from human aging physiology [31,51]. To address this, human skin equivalents (HSE) have been previously established as valuable models in the study of skin and aging [4,8,12,14,99,100,101,102,103,104,105,106,107,108]; however limitations remain. Of special relevance, loss and dysregulation of hypodermal adipose is implicated in physiological aging [98,109,110], and aging-associated diseases, including lipoatrophy [111] associated with insulin-resistant diabetes mellitus [112]. This dysregulation is poorly captured in current HSE. To address this, we have developed a robust and reproducible HSE that includes adipose and vascular components (AVHSE). This methodology builds off of previous studies [62,113,114,115,116] and provides a model to study crosstalk between adipose, vascular, stromal, and epithelial components of skin in the context of aging. Furthermore, this model is tissue-scale, stable for long culture durations (experiments described were 8 weeks, 16-week cultures have been performed using similar methods), and suitable for aging studies. Other researchers have reported that when skin models are cultured with adipose tissue, after 2 weeks of culture, there was epidermal disintegration and that 7 days is enough time at ALI to produce a fully functional skin equivalent [64]. Although we did not directly compare skin equivalents without adipose to AVHSEs here or directly compare culture timepoints, we have not observed any obvious changes in epidermal coverage compared to our previous work in vascularized human skin equivalents that do not contain a subcutaneous adipose compartment [59]. While the model is customizable to study the effects of intrinsic and extrinsic aging factors, as a test case we have demonstrated suitability for studies in UVA photoaging due to the strong literature base of both in vitro and in vivo studies available for comparison. Finally, we demonstrated the accessibility of the model for both molecular (e.g., ELISA) and morphological studies (e.g., volumetric analysis of cell organization).

A key aspect of any HSE model is a differentiated and stratified epidermis. In this study, N/TERT-1 keratinocytes [68] were used to generate skin epidermis, as previously completed [31,35,59]. Importantly, N/TERTs are a suitable and robust substitute to primary keratinocytes, which have disadvantages including limited supply, limited in vitro passage capabilities, and donor variability [35]. HSEs generated with N/TERT keratinocytes demonstrate comparable tissue morphology, appropriate epidermal protein expression, and similar stratum corneum permeability when compared to HSEs generated with primary keratinocytes [31,35]. Similar to prior models, we demonstrate AVHSEs appropriately model the skin epidermis with correct localization of involucrin (a stratum corneum marker) and cytokeratin 10 (suprabasal early differentiation marker) [1,38] (Figure 4). Furthermore, volumetric imaging and automated analysis allows for epidermal thickness to be robustly calculated. AVHSE present with median epidermal thicknesses within 90–100 µm, similar to values in both prior in vitro studies 100–200 µm [117] and in vivo optical coherence tomography imaging of adult skin 59 ± 6.4 to 77.5 ± 10 µm [118]. Consistent with prior in vitro and in vivo results showing UVA wavelengths predominantly impact dermal rather that epidermal layers [119,120], UVA photoaging resulted in no observable changes in epidermal thickness or expression of differentiation markers in AVHSE (Figure 4).

In the dermis and hypodermis, skin is highly vascularized with cutaneous microcirculation playing important roles in thermal regulation and immune function [98,121]. Many prior HSE models have not included a vascular component [31,34,35,43,63]; however, there is increasing recognition of its importance [1,4,40,47,48,122,123]. In the present work, we used collagen IV as a marker of the vascular basement membrane, enabling the automated segmentation and mapping of a vascular network within AVHSEs. Importantly, volumetric quantification of the vasculature was performed with imaging after tissue clearing; however, these techniques are possible with uncleared images as well with some limitations [59]. The vascular VF of AVHSEs is lower than in vivo dermis (~3% compared to 20.0 ± 5.0 to 40.3 ± 2.4% measured by OCT at four positions in the arm [124]), but prior work has shown this is tunable by using different cell seeding conditions [79]. Optimizing the VF may be more involved in the AVHSE, since the ratio of adipose and vascular cells has been shown to be important in regulating tissue morphology [113]. Thus, ratio of adipose and vascular cells would need to be optimized for new cell and collagen densities. Adipose tissue is densely vascularized [113,125,126], and the ability of adipocytes to generate lipid droplets and adipokines in the presence of endothelial cells is important to replicate the in vivo environment [114]. Previous work has shown that in co-culture of endothelial cells (ECs) and mature adipocytes can lead to dedifferentiation of mature adipocytes [126], but in homeostatic cultures, ECs and adipocyte crosstalk is important. Through soluble factor release, Ecs regulate lipolysis and lipogenesis, and adipocytes regulate vasodilation and contraction [64,126]. Secretion of adipokines by adipocytes aids vascular formation and adipose tissue stability [114,126]. In prior work, Hammel and Bellas demonstrated that 1:1 is the optimal ratio for vessel network within 3D adipose [113], and we matched the 1:1 cell ratio in the present work.

Quantification of vessel diameter in the Hammel and Bellas study shows that a 1:1 ratio of adipocytes to endothelial cells gives an average vessel diameter of ~10 µm [113], our work similarly finds a median vessel diameter of ~6 µm. Importantly, these data are within the range of human cutaneous microvascular of the papillary dermis (4 to 15 µm [121]). We did not observe morphological changes of VF and diameter within the vasculature due to photoaging. While it is established that chronic UVA exposure can contribute to vascular breakdown [97,127], the duration of our studies may be too short to see this effect in diameter and VF (1 week vs. lifetime UV exposure in people over 80 [3,97]). However, photoaging did induce an increase in diffusion length (R_k_), in this case defined as the distance from the vasculature that covers 90% of the construct; higher values correspond to lower vascular density. Values presented here match previous studies of vascularized collagen [79]. R_k_ of the vascular network for both control and photoaged samples was within the range of 51–128 µm, lower than the frequently cited 200 µm diffusion limit for supporting a cellular tissue [128]. These findings conflict with studies of acute UV exposure in skin, which show stimulation of angiogenesis [3,129]. It has been proposed that UV light exposure may improve psoriasis by normalizing disrupted capillary loops through upregulation of VEGF by keratinocytes [121]. The AVHSE model could be used to more thoroughly test the effects of UV light and other molecular mechanisms it induces in future studies.

Furthermore, we observed vasculature colocalized with the lipid droplet BODIPY staining (Figure 2), indicating recruitment of the vascular cells to the hypodermis. Importantly, the vascular networks in prior studies and the present AVHSE are self-assembled. While there are advantages to self-assembly, especially the simplicity of the method, it is important to note the limitations. Cutaneous microcirculation in vivo has a particular anatomical arrangement with two horizontal plexus planes, one deep into the tissue in the subcutaneous fat region and one just under the dermal-epidermal junction [121,130]. Between these two planes are connecting vessels running along the apicobasal axis that both supply dermal tissues with nutrients and are an important part of thermoregulation [121,130]. Although the AVHSEs presented here have reasonable vascular densities they do not recapitulate this organization. While not covered in this work, future studies could incorporate layers of patterned or semi-patterned vasculature [128] to more closely match the dermal organization, depending on the needs of the researcher.

This photoaging model did demonstrate impacts to the hypodermis. Volumetric imaging of BODIPY, which stains lipid droplets [113], was used as a measure of adiposity. While small reductions in the morphological parameters (adipose thickness and lipid VF) were observed, they were not significant, suggesting there was not large-scale apoptosis or other cellular loss. However, there was a significant decrease in the intensity of BODIPY staining, indicating decreased lipid levels (Figure 6). This is consistent with photoaging of excised human skin showing that UV exposure decreases lipid synthesis in subcutaneous fat tissue [96]. We further collected culture supernatant and tested for the presence of adiponectin, IL6, and MMP-1. The data collected through ELISA (Figure 3) show that this AVHSE model secretes both adiponectin and IL6, which are also present in native skin and both considered important adipokines [62,126,131,132]. Elevated serum adiponectin levels are linked to anti-inflammatory effects in humans [131,132], and centenarians (a model of healthy aging) have elevated levels of adiponectin [131]. Decreased adiponectin has previously been associated with photoaging in both excised human skin that was sun-exposed compared to protected skin and in protected skin that was exposed to acute UV irradiation [24]. Conversely, IL6 is a key factor in acute inflammation in skin, and has been shown to regulate subcutaneous fat function [96,133]. In prior studies of photoaging, IL6 has demonstrated an increase after UVA irradiation in monolayer fibroblast cultures [134] and excised human skin [96,133]. IL-6 is released after UV irradiation and has been linked to decreased expression of adipokine receptors and mRNA associated with lipid synthesis [24], decreases in lipid droplet accumulation [96], and enhanced biosynthesis of MMP1 [134,135]. However, after one week of photoaging, we did not observe an increase in IL-6 or MMP-1 via ELISA (Figure 3).

The absence of changes in IL-6 and MMP-1 expression but decreases in lipid accumulation and adiponectin are not expected results, but they could be due to methodology differences in UVA exposure. We determined our UVA dose and exposure based on the literature values [18,23,24,26,50,84,85]. The dose used here was 0.45 ± 0.15 mW/cm^2^ with exposure for 2 h daily for 7 d; this converts to approximately 3.24 J/cm^2^ per day and a total of 22.68 J/cm^2^. Many studies do not report exposure time and/or present ambiguous timepoints. This compounded with the practice of using doses based on sample pigmentation and broad definition of UVA wavelengths may be contributing to the differences in IL-6 and MMP-1 expressions compared to prior work. While not addressed in the present study, the AVHSE culture platform is suited for future studies investigating the specific molecular mechanisms associated with altered wavelength(s), dosing, and durations.

Unexplored in this study is the mechanics of the AVHSE, and the impact of photoaging on mechanics. Acting as a defense against mechanical trauma is a key function of the skin in vivo [1], the mechanical properties that enable this are highly dependent on the structure and composition of the tissue [136]. Importantly, the collagen density in the AVHSE model is 3 mg/mL, much lower than in vivo densities [137,138]; this difference is likely accompanied by dramatically reduced mechanical strength of AVHSE compared to native skin. Increasing the collagen density to physiologically relevant levels and directly quantifying AVHSE mechanics will be an important aspect of future studies [136]. Importantly, higher collagen densities are possible through a variety of techniques, including dense collagen extractions [81] and compression of the collagen culture [41]. In addition to density, the anisotropic collagen organization of native dermis is a key factor in mechanics. It has long been understood that dermal collagen has a preferential orientation, and that the mechanical properties of skin differ parallel or perpendicular to that orientation [136,139]. In these studies, collagen alignment was not controlled and likely has no global alignment [140]; future studies that included collagen orientation could leverage a range of previously established techniques depending on the specific goals [141]. In the context of photoaging, these mechanical studies are especially relevant. Decline of collagen density is an important aspect of skin aging, correlating with skin elasticity and wound healing [4,5,6,7,11,17,142]. Furthermore, photoaging increases the expression of MMPs and decreases collagen synthesis [23,24,25], resulting in fragmentation and disorganization of the dermal extracellular matrix [18]. Future studies that measure and control the mechanics of AVHSE will be important for fully characterizing the model and more closely matching the physiology of native skin.

There are additional limitations of the AVHSE model presented. Although we have presented a skin model that is closer to both anatomy and biology of human skin in comparison to past HSEs, we have not modeled skin fully through inclusion of other features of in vivo skin, such as immune and nerve components. Including a functional immune system is important in understanding autoimmune diseases, cancer, wound healing, and decline of immune function in aged skin [4,98]. Additionally, neuronal cell inclusion will allow for modeling of sensory processes necessary for grafting and modeling of skin disorders associated with nerve dysregulation [98]. Furthermore, while the cell lines used in this study were chosen for their low cost and accessibility, primary cells or populations differentiated from induced pluripotent stem cells (iPSCs) would more closely match the physiology in vivo. While changing cell populations would likely require some adjustment to the culture system, we have previously demonstrated that cell types can be replaced with minimal changes [59]. We model epidermis, dermis, and hypodermis here, but we do not model the depth that is present in thick skin tissue. As nutrient and waste diffusion in tissues is limited to ~200 µm [128], thick tissues will likely require perfusion to maintain throughout culture. Vasculature in thicker skin has higher diameters, especially in the lower dermis and hypodermis, these can be up to 50 µm [121], and thick skin models may benefit from patterning larger vessels. Furthermore, the AVHSE method was demonstrated with low serum requirements; while serum was used for initial growth, the cultures are maintained for weeks without serum. Serum replacements during the growth phase could potentially provide a chemically defined xeno-free culture condition in beginning culture stages for greater reproducibility and biocompatibility.

The presented AVHSE model provides unique capabilities compared to cell culture, ex vivo, and animal models. Excised human skin appropriately models penetration of dermatological products but there is limited supply and high donor variability [143]; replacing excised human skin with animal models or commercially available skin equivalents is limited by the differences, such as varying penetration rates, lipid composition, lipid content, morphological appearance, and healing rates [143,144]; and cost and limitations of customization are additional factors. AVHSE can be cultured using routinely available cell populations, is cost effective, and is customizable for specific research questions. Furthermore, the model is accessible for live imaging, volumetric imaging, and molecular studies, enabling a wide range of quantitative studies. The current work focused on AVHSE as a research tool, but similar techniques could be further developed for the development of grafts. Grafting would require addressing many of the structural and biological limitations noted above, and modifications to address host immunity issues. In conclusion, we have demonstrated AVHSEs as a research platform with regards to photoaging effects, but expansions of this model could be utilized for clinical skin substitutes [73], personalized medicine, screening of chemicals/cosmetics, drug discovery, wound healing studies [73,144], and therapeutic studies [62].

## Figures and Tables

**Table 1:** Media used for 2D and 3D culture.

Cell Line or Culture Period	Recipe	Notes	Corresponding Timepoint
N/TERT 1	K-SFM base media 1% P/S Bovine Pituitary Extract (BPE) [25 µg/mL] Epidermal Growth Factor (EGF) [0.2 ng/mL] CaCl_2_ [0.3 mM]	Media recipe based off of these references [31,68]. BPE and EGF are from the K-SFM supplement kit.	Maintenance culture
HMEC1	MCDB131 base media 10% FBS 1% P/S L-Glutamine [10 mM] Epidermal Growth Factor (EGF) [10 ng/mL] Hydrocortisone [10 ug/mL]	Media recipe as recommended by manufacturer.	Maintenance culture
Human Dermal Fibroblasts	DMEM HG base 5% FBS 1% P/S	Media used for short term expansion in 2D. For longer expansion, use the manufacturer recommendation.	Maintenance culture
ASC52telo	Mesenchymal Stem Cell Basal Medium 2% MSC supplement L-Alanyl-L-Glutamine [2.4 mM] G418 [0.2 mg/mL]	MSC Basal Medium is from ATCC (ATCC PCS-500-030); To make the complete medium the MSC growth kit (ATCC PCS-500-040) is added. MSC supplement from the growth kit contains: 2% FBS, 5 ng/mL rhFGF basic, 5 ng/mL rhFGF acidic, 5 ng/mL rhEGF.	Maintenance culture
Adipogenesis Differentiation media	DMEM/HAM’s F12 base media 3% FBS 3-isobutyl-1-methyl-xanthane (IBMX) [250 µM] Indomethacin [10 µg/mL; 28 µM] Insulin [5 µg/mL] Dexamethasone [1 µM] D-pantothenate [34 µM] Biotin [66 µM]	Media recipe is based on prior work [71,72]. IBMX, Insulin, and Dexamethasone stocks stored at −20 °C. Indomethacin, D-pantothenate, and Biotin stocks stored at 4 °C.	3 weeks prior to dermal seeding. 1 week for 2D culture and 2 weeks for 3D culture.
Adipocyte Maintenance Media (serum free)	DMEM/HAM’s F12 base media Insulin [5 µg/mL] Dexamethasone [1 µM] D-pantothenate [34 µM] Biotin [66 uM]	This media blend is not used by itself for AVHSE culture, but it is used to make dermal submersion media. Adipocyte maintenance media is adipogenesis differentiation media without IBMX or Indomethacin (a PPARγ agonist) [72]	Used indirectly for Dermal Submersion media.
Dermal Submersion (DS)	1:1 Serum Free Adipocyte Maintenance media and serum free HMEC1 media *Aliquot supplement:* 3% FBS *Daily supplements:* L-Ascorbic Acid [100 µg/mL], VEGF [2 ng/mL]	Dermal submersion media is half adipocyte maintenance media and half HMEC1 media with supplement changes. Media prepared serum-free and used as base for ESM and AVHSE media.	During week 4 of culture: dermal cells are seeded and dermis is maturing.
Epidermal Seeding and maturation media (ESM)	Dermal submersion media with CaCl_2_ [1.44 mM] *Aliquot supplement:* 1% FBS *Daily supplements:* L-Ascorbic Acid [100 µg/mL]	Media used for addition of N/TERT1s, shares base with DS and AVHSE media.	During week 4 of culture: epidermal cells are seeded and maturing.
AVHSE media	Dermal submersion media with CaCl_2_ [1.44 mM] *Daily supplements:* L-Ascorbic Acid [100 µg/mL], Selenium (sodium selenite) [30 nM]	AVHSE media is serum free. L-ascorbic acid is important for collagen synthesis by fibroblasts, collagen stability, vessel wall integrity and barrier function [73,74,75,76,77,78].	~4 weeks into whole culture and through culture endpoint. Media is used for ALI.

**Table 2:** Staining sequence, antibodies, and blocking buffer used.

Staining Sequence			
Stain/Imaging Phase	Staining/Processing Used	Imaging Orientation	
1. Epidermal	Cytokeratin 10, Involucrin, DRAQ7	Apical (epidermal)	
2. Dermal Vasculature	Collagen IV	Basal (hypodermis)	
3. Adipose	BODIPY	Basal (hypodermis)	
4. Post-clearing	(Methanol dehydration, methyl salicylate clearing)	Basal (hypodermis)	
Epidermal Staining			
Antibody/Stain	Information and Source	Concentration	Notes
DRAQ 7	Cell Signaling;	[1:250]	Nuclear marker
Cytokeratin 10			Suprabasal epidermal marker
Primary	Cytokeratin 10 (DE-K10) mouse IgG, supernatant. Santa Cruz; sc-52318		
Secondary	Goat Anti-Mouse IgG (H&L), DyLight™ 488. Thermo Scientific; 35502 (1 mg/mL)	[1:500]	
Involucrin			Stratum Corneum, terminal differentiation marker [32]
Primary	Involucrin rabbit polyclonal IgG. Proteintech; 55328-1-AP (30 µg/150 µL)		
Secondary	Anti-Rabbit IgG (H&L) (GOAT) Antibody, DyLight™ 549 Conjugated. Rockland Immunochemicals; 611-142-002	[1:500]	
Dermal Vasculature Staining			
Collagen IV			Vascular basement membrane
Primary	Collagen IV rabbit polyclonalProteintech; 55131-1-AP	[1:500]	
Secondary	Anti-Rabbit IgG (H&L) (GOAT) Antibody, DyLight™ 549 Conjugated. Rockland Immunochemicals; 611-142-002	[1:500]	
Adipose Staining			
BODIPY	Difluoro{2-[1-(3,5-dimethyl-2H-pyrrol-2-ylidene-N)ethyl]-3,5-dimethyl-1H-pyrrolato-N}boron; dissolved in 200 proof EtOH, CAS: 121207-31-6; Aldrich; 790389	[2 µM]	Mature adipocyte marker
Clearing			
Methanol	CAS: 67-56-1	4 baths, 10 min each	For sample dehydration.
Methyl Salicylate	CAS: 119-36-8	4 baths, 5 min each	For sample clearing
Blocking Buffer Recipe			
Reagent		Amount	
ddH_2_O		450 mL	
10 × PBS		50 mL	
Bovine Serum Albumin (BSA)		5 g	
Tween 20		0.5 mL	
Cold water Fish Gelatin		1 g	
Sodium Azide (10% Sodium Azide in diH_2_O)		5 mL (0.1% final concentration)	
All exposure for stains and antibodies: 48 h, stationary, 4 °C			

## Data Availability

The data that support the findings of this study are available from the corresponding author, JTM, upon reasonable request.

## Supplementary Materials

The following supporting information can be downloaded at: https://www.mdpi.com/article/10.3390/biom12121828/s1, Figure S1: UVA Photoaging Setup.
