# Supplementary material for: Development of a Vascularized Human Skin Equivalent with Hypodermis for Photoaging Studies
Source: Biomolecules. 2022 Dec 7;12(12):1828. doi: 10.3390/biom12121828 (PMC9775308; doi:10.3390/biom12121828)
Supplement: Supplementary file 1 [file biomolecules-12-01828-s001.zip › biomolecules-2020440-supplementary.pdf]

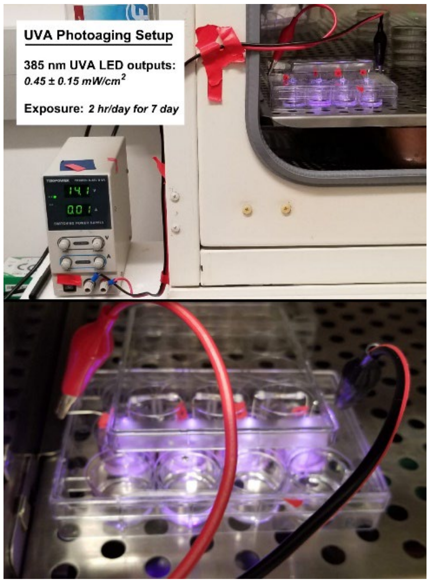

Figure S1: UVA Photoaging Setup. UVA exposure was completed by drilling out a well plate lid and inserting UVA LEDs. Each LED had an output of 0.45 ± 0.15 mW/cm^2. AVHSEs were exposed daily for 2 hours, for 7 days.
